# Emergency department imaging utilization post-transcatheter aortic valve replacement: single institution 7-year experience

**DOI:** 10.1007/s10140-024-02228-y

**Published:** 2024-04-23

**Authors:** Eva Chau, Andy Mew, Kaustav Bera, Sirui Jiang, Nikhil Ramaiya, Robert Gilkeson

**Affiliations:** 1 University of Pittsburgh School of Medicine, Pittsburgh, OH USA; 2 Case Western Reserve University School of Medicine, Cleveland, OH USA; 3 Department of Radiology, University Hospitals Cleveland Medical Center, Cleveland, OH USA

**Keywords:** Transcatheter aortic valve replacement, Computed tomography, Ultrasound, Magnetic resonance imaging, Nuclear imaging, Emergency department Readmissions, Complications

## Abstract

**Purpose:**

This study aims to highlight presentations, acute findings and imaging phenotypes of patients presenting to the emergency department (ED) within 30 days of a transcatheter aortic valve replacement (TAVR).

**Methods:**

A retrospective review of patients diagnosed with aortic valve disease who underwent a TAVR between Jan 2015 and Nov 2021 at a large academic medical center was completed. From an initial 1271 patients, 146 were included based on their presentation to the ED within 30 days post-TAVR procedure. Patient data, including ED presentation details and imaging results, were recorded and de-identified.

**Results:**

Of the 146 post-TAVR patients, there were 168 ED visits within 30 days. The median time to ED after TAVR was 12 days. Respiratory symptoms were the most common complaint (27%). Neurological (23%) and cardiovascular symptoms (18%) followed. Cross-sectional imaging was conducted 250 times across visits, with an average of 1.7 scans per patient. CTs were most frequently used, followed by ultrasounds, especially echocardiograms and duplex extremity vasculature ultrasounds. 30.1% of patients had acute findings from imaging. Specific findings included heart failure (5.5%), access site complications (5.5%), pneumonia (5.5%), intracranial pathologies (3.4% for strokes and 0.7% for hematoma), and pleural effusion (3.4%). Echocardiograms and CTA chest were most associated with significant acute findings.

**Conclusion:**

Our study highlights the vital role of early and accurate imaging in post-TAVR patients within 30 days post-procedure. As transcatheter approaches rise in popularity, emergency radiologists become instrumental in diagnosing common post-procedural presentations. Continued research is essential to devise post-discharge strategies to curtail readmissions and related costs. Proper imaging ensures prompt, effective care, enhancing overall patient outcomes.

## Introduction

In the turn of the century, transcatheter aortic valve replacement (TAVR) has gradually overtaken surgical aortic valve replacement (SAVR) as the leading intervention for patients with aortic stenosis.^1^ While the rates of procedural success is high in the short term for TAVRs, long term risks of readmission continue to pose a challenge to patient outcomes and health system costs.^2^ In 2015, data from the Society of Thoracic Surgeons/American College of Cardiology (STS/ACC) Transcatheter Valve Therapies Registry linked with patient-specific administrative claims data from the Centers for Medicare & Medicaid Services (CMS) found that any-cause readmission rates following TAVR as high as 17.4% within the first 30 days.^3^ While there appears to be a plethora of research regarding peri-procedural TAVR complications such as device landing zone rupture, coronary artery obstruction and valve embolization, literature regarding the complications in the 30 day period after discharge from the index admission is less definitive.^4^ As the various imaging modalities ordered at patient presentation are imperative in quickly and accurately identifying the presence of complications in the post cardiac valve surgeries patient population^5^, this study aims to shed light on the trends in presentation common to the post TAVR population, specifically within the first 30 days post-procedure.

Given the advanced age of most patients undergoing TAVR and the sizable and growing quantity of TAVR procedures carried out annually in the US (72,991 in 2019)^1^, emergency radiologists will be the first to interpret various ED imaging and must be prepared for the growing number and imaging nuances of post-TAVR patients. Because the Center for Medicare and Medicaid Services continues to utilize readmission as a quality metric that affects reimbursement, it is imperative for emergency personnel to be familiar with trends of ED presentation especially in the critical 30 day post-procedural period. Additionally, with the growing importance of cognizant healthcare spending, we believe it necessary to simultaneously explore the financial impact of imaging in this patient population.

The goal of this study is to first inform radiologists of common imaging needed in the post-TAVR population within the 30 day post-procedural period and common presenting symptoms and acute imaging findings to be on the lookout for. We will then analyze the subsequent costs of the necessary imaging.

## Materials and methods

**Fig. 1:**
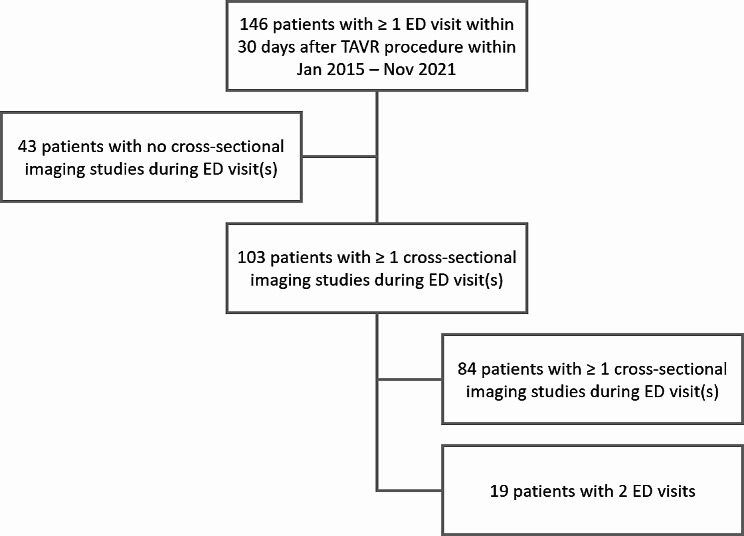
Study population

After retrospective review of the hospital electronic medical record (EMR), patients with a diagnosis of aortic valve disease that were evaluated for and received a TAVR at a large academic medical center between Jan 2015 and Nov 2021 were included in this study. The study protocol was approved by the institutional review board of our institution, and all patients have provided prior informed consent for the imaging that they received. The study group was made up of an initial 1271 patients prior to inclusion and exclusion criteria. Our group of interest included patients who presented to the ED within 30 days of their TAVR procedure. With this criteria, 146 patients were included in our study (Fig. 1).

Information related to patient ED presentation was recorded and de-identified. This included date of presentation, length of stay, reason for presentation, indication for imaging, imaging obtained, imaging findings, and any resulting diagnoses. This was confirmed via supporting evidence located in physician notes in the EMR and were further categorized based on malignancy when relevant.

A radiologist-in-training in collaboration with a fellowship trained radiologist (21 years of experience) then reviewed cross-sectional imaging findings to confirm their correlation with the final diagnoses that were documented by the ED team or primary team. Cross-sectional imaging obtained in the ED including computed tomography (CT), magnetic resonance imaging (MRI), ultrasound (US), image guided procedures and nuclear medicine ventilation- perfusion (VQ) scans, were recorded.

## Results

### Patient demographics

Our study population consisted of 146 patients with a diagnosis of aortic stenosis and an ED presentation within 30 days of their TAVR procedure. The demographics of our study were made up of mostly geriatric patients with an average age of 79.7 (median 79.7 years, interquartile range 72.5–86.6). Male patients made up a larger portion of the study population at 61% compared to the female patients at 39%.

**Table 1:** Patient Demographics

Characteristic	Value
Total number of patients	146
Age (y), mean ± SD	78.6 ± 10.1
Age (y), median	79.7
Range	40.6–95.1
Interquartile range	72.5–86.6
Sex	
Female	57 (39%)
Male	89 (61%)
ED visits	
Total ED visits	168
Number of admissions	117
Median days after TAVR for presentation	12
Range (days)	2–30
Interquartile range (days)	6–21
Hospital stay	
Median length of hospital stay	2
Range (days)	0–34
Interquartile range (days)	0–4
Number of deaths during admission	7

Among our 146 patients, there was a collective total of 168 ED visits within 30 days of the TAVR procedure. 124 patients (85%) visited the ED once and 22 (15%) visited the ED twice within this time frame. Among these 168 total ED visits, 117 (70%) resulted in admission to an inpatient service. The median time from the TAVR procedure to ED presentation was 12 days, with a range of 2 to 30 days and an interquartile range of 6 to 21 days. The median length of hospital stay upon ED presentation was 2 days, with a range of 0 to 34 days and an interquartile range of 0 to 4 days. Of all patients who presented to the ED, 7 (4.8%) passed away during their hospital stay (Table 1).

The common presenting complaints were respiratory symptoms, mentioned in 45 of the 168 total visits (27%), most notably dyspnea (*n* = 43) followed by cough (*n* = 3). Thereafter, neurological symptoms were reported in 38 instances, comprising 23% of all visits. Common neurologically related complaints encompassed falls (*n* = 15), syncope (*n* = 8), dizziness (*n* = 7), altered mental status (*n* = 7), and lightheadedness (*n* = 7). Cardiovascular symptoms, reported in 30 cases, ranked third and made up 18% of visits. Within this category, chest pain was the predominant presenting symptom (*n* = 21), followed by palpitations (*n* = 4). Additional presenting symptoms included vascular complaints in 26 patients (15%), abdominal symptoms in 24 patients (14%), constitutional symptoms in 22 patients (13%), and access-related symptoms in 15 patients (9%) (Table 2).

**Table 2:** Presenting symptoms*

System	# of Patients With Symptoms	% of Visits (*n* = 168)	Common Symptoms*
Respiratory	45	27%	dyspnea (43), cough (3)
Neurologic	38	23%	fall (15), syncope (8), dizziness (7), altered mental status (7), lightheadedness (7)
Cardiac	30	18%	chest pain (21), palpitations (4)
Vascular	26	15%	leg swelling (9), hypotension (6), leg pain (5)
Abdominal	24	14%	Abdominal pain (8), GI bleed (7), nausea (4), vomiting (3)
Constitutional	22	13%	weakness (16), fever (5), fatigue (3)
Access Site	15	9%	site bleed (10)

*some visits had multiple presenting symptoms. Common symptoms are not all-inclusive of all presenting symptoms

On chart review, the most commonly cited indications for imaging during the ED visit or during their subsequent readmission included dyspnea, fall, chest pain, head injury/ trauma and syncope. Neurologic indications for imaging were the most common in this population (*n* = 52) followed by vascular indications (*n* = 37) and cardiac indications (*n* = 25) (Table 3). While never mentioned as a sole indication for cross-sectional imaging, post-TAVR status was often included alongside other indications.

**Table 3:** Common cross-sectional imaging indications*

System Indication	# of Imaging Studies With Indication	Common Indications	Common Imaging Obtained
Neurologic	52	fall (15), head trauma (10), syncope (10), altered mental status (6), rule out stroke (2), encephalopathy (2), headache (2), confusion (2), subdural hematoma (2)	CT Head wo cont (22), CT C-spine (10), MRI Brain wo cont (4), CT facial bones (2), Echocardiogram (2)
Vascular	37	pulmonary embolism rule out (9), anticoagulated (8), elevated D-dimer (4), hypotension (3), hemoglobin drop (3), rule out bleed (3), leg pain at rest (3)	CTA chest (11), CT Chest Abd Pelv wo cont (2), CT Abd Pelv wo cont (7), CT head wo cont (5), duplex lower extremity venous US (5), duplex lower arterial US (3), CT C-spine (2), lower venous duplex US (2), NM Lung scan (2)
Cardiac	25	chest pain (12), pericardial effusion (4), congestive heart failure (3), rule out pericardial effusion (2), atrial fibrillation (2), post-pacemaker placement (2)	CTA Chest (8), Echo (15)
Respiratory	24	dyspnea (20), cough (2), hypoxia (2)	CTA chest (10), Echo (5), CT Chest wo cont (4), NM Lung Scan (3)
Abdominal	21	abdominal pain (6), nausea (4), vomiting (4), GI bleed (4), abdominal/pelvic hematoma (3)	CT Chest Abd Pelv wo cont (9), CT Abd pelv w cont (2), US Abd (2), US gallbladder (2)
Access Site	16	pseudoaneurysm eval (6), site bleeding (4), site swelling/edema (2), groin pain (2), stenosis at left common femoral artery (2)	duplex lower extremity venous US (8), Duplex lower extremity arterial US (3), US doppler (3)

*Many imaging studies listed multiple indications

A total of 250 cross-sectional imaging examinations were obtained across all ED visits for the included 146 patients, with an average of 1.7 examinations per patient (Table 4). The most common imaging modality utilized was CTs (*n* = 129). CT examinations ordered include CT head (*n* = 39), CTA Chest (*n* = 26) CT abdomen and pelvis (A/P) with contrast (*n* = 16), CT chest without contrast (*n* = 24) and C-spine without contrast [11]. Ultrasounds were the second most common type of cross-sectional imaging ordered (*n* = 101). The 2 most common ultrasound modalities were echocardiograms (*n* = 47) and duplex extremity vasculature ultrasounds (*n* = 40). Other modalities also include MRI brain (*n* = 12), nuclear imaging (*n* = 7), and fluoroscopy (*n* = 1).

**Table 4:** Common imaging obtained

Imaging Modality	# of Studies	% of Studies	Common Imaging Ordered*
CT	129	51.6%	Head CT without Contrast (39), Chest Angio Chest (26), Abdomen Pelvis with Contrast (16), CT Chest without Contrast (12), C-spine Without Contrast (11)
Ultrasound	101	40.4%	Echocardiograms (*n* = 47), Duplex Extremity Vasculature Ultrasounds (*n* = 40)
MRI	12	4.8%	Brain MR without contrast (4)
Nuclear Imaging	7	2.8%	NM Ventilation Perfusion Lung Scan (4)
Fluoroscopy	1	0.4%	Evaluation of Swallowing Function (1)
Total	250		

*Common imaging ordered not all-inclusive of all imaging ordered under each modality

There were 47 acute findings overall from the imaging that were obtained. Of note, 3 patients had 2 acute findings each. In total, 44 of the 146 patients (30.1%) were found to have acute findings.

**Fig. 2:**
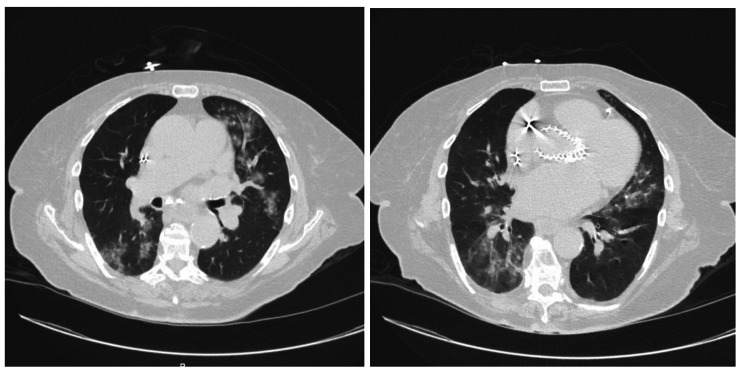
75 year old female with history of hyperlipidemia presenting with nausea and generalized weakness. Axial CT images in lung window demonstrate bilateral multifocal nodular and patchy ground-glass opacities consistent with infectious pneumonia

**Fig. 3:**
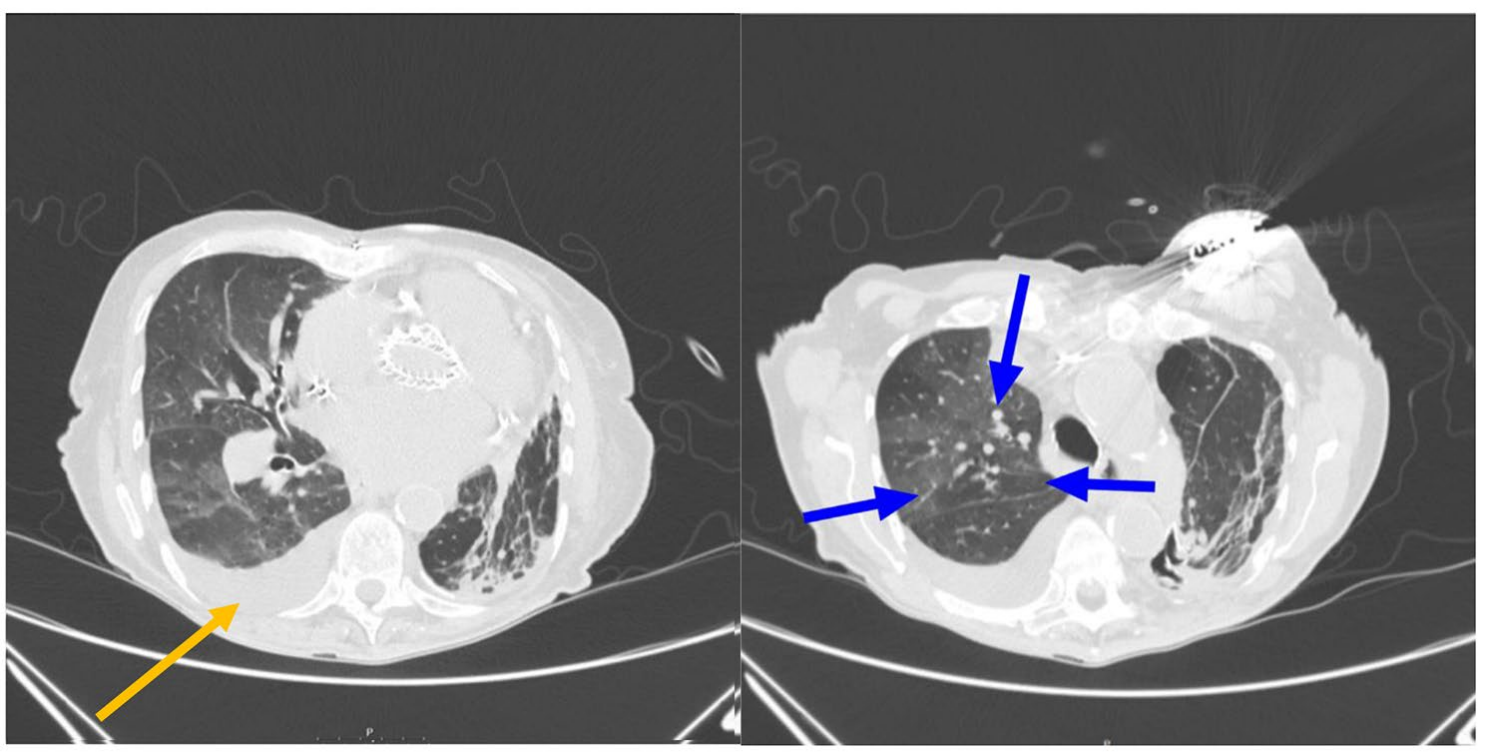
84 year old female with history of Monckeberg’s arteriosclerosis presenting with shortness of breath. Axial CT images in lung window shows (**A**) pleural effusion (orange arrow) and (**B**) interlobular septal thickening and ground glass opacities (blue arrows) representing pulmonary edema

Common imaging findings from chest imaging included 7 cases of pneumonia and 4 pleural effusions. Presenting symptoms varied widely from patient to patient for pneumonia, with no clear trend (Fig. 2). The most common presenting symptom was shortness of breath for patients with pleural effusions, present in 4 of 5 patients (Fig. 3). Of note, 1 case of pleural effusion was found on abdominal imaging.

**Fig. 4:**
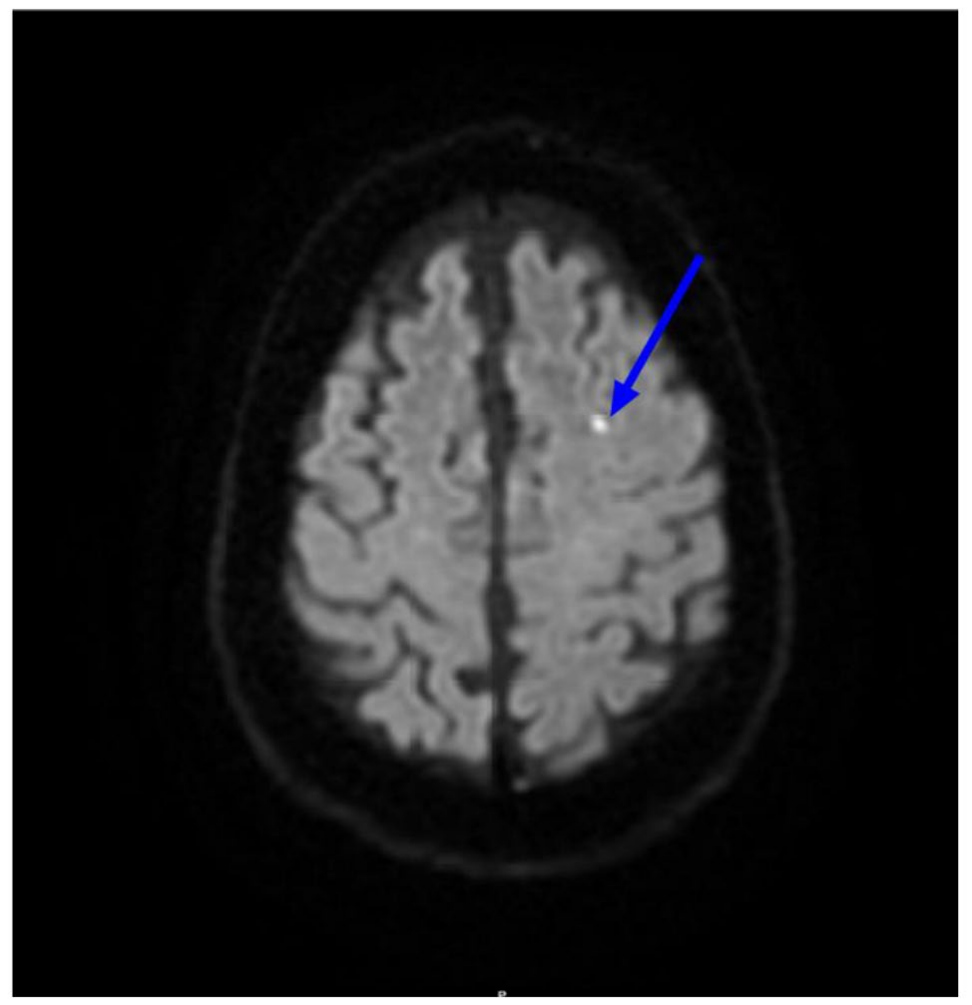
71 year old with a history of pernicious anemia. Presenting with fatigue and left sided temporal headache. Axial Diffusion weighted (DWI) MRI imaging of the brain demonstrates small focus of restricted diffusion in left frontal lobe consistent with acute or subacute infarct

**Fig. 5:**
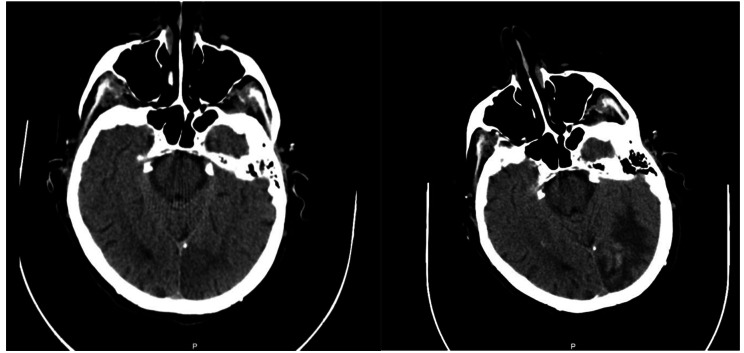
81 year old female with a history of peripheral vascular disease presenting with toe pain in bilateral feet. (**A**) Axial CT head 26 days prior to ED visit demonstrating loss of gray-white differentiation in the medial left occipital lobe, consistent with acute to subacute left PCA territory infarction. (**B**) On presentation Axial CT head demonstrates evolution of a left occipital subacute infarction, larger and more discrete appearing than previously, also now with petechial hemorrhage

Head and neck imaging findings included 4 cases of stroke and 1 brain hematoma. Altered mental status was the predominant presenting symptom, which was found in 4 of 5 patients (Figs. 4 and 5).

**Fig. 6:**
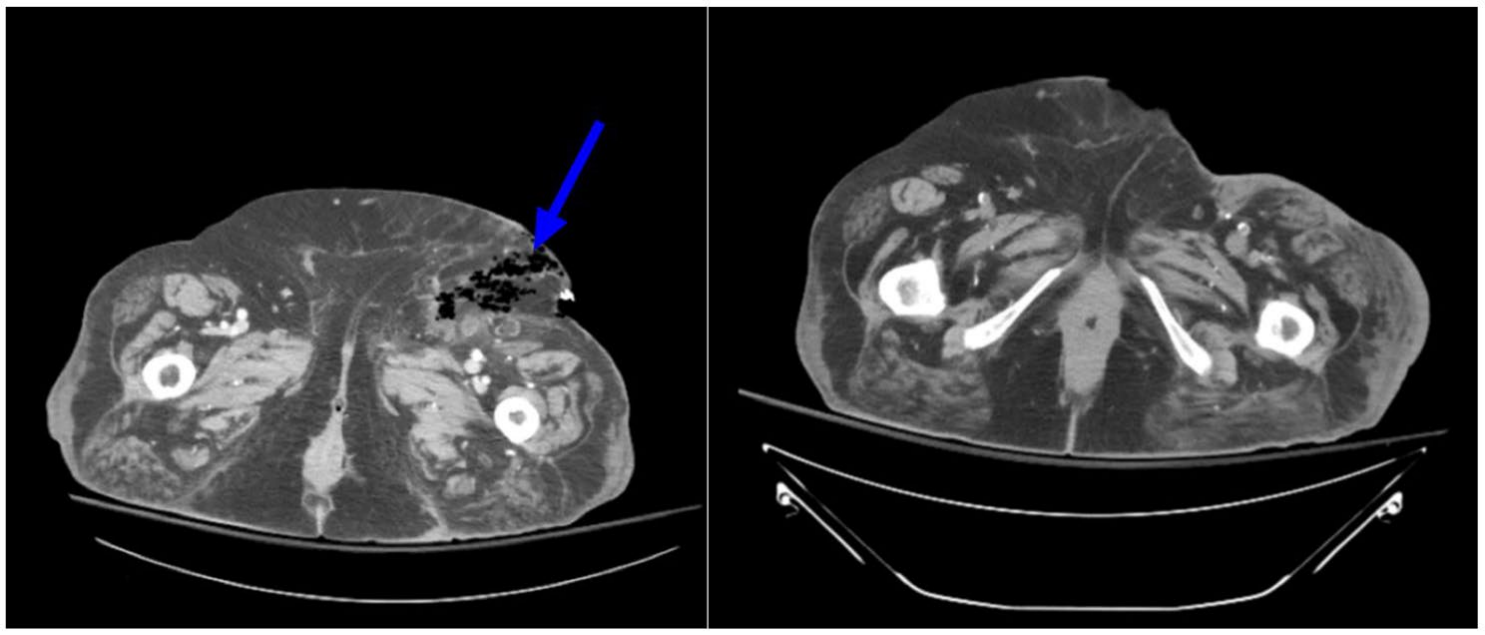
69 year old female presenting with infected groin wound. Axial CT images of the pelvis in soft tissue window demonstrate (**A**) fat stranding and foci of air in the left lower abdominal wall, demonstrating necrotizing fasciitis. (**B**) Post treatment CT pelvis in soft tissue window resolution of necrotizing fasciitis status-post debridement and repair of the left inguinal area

**Fig. 7:**
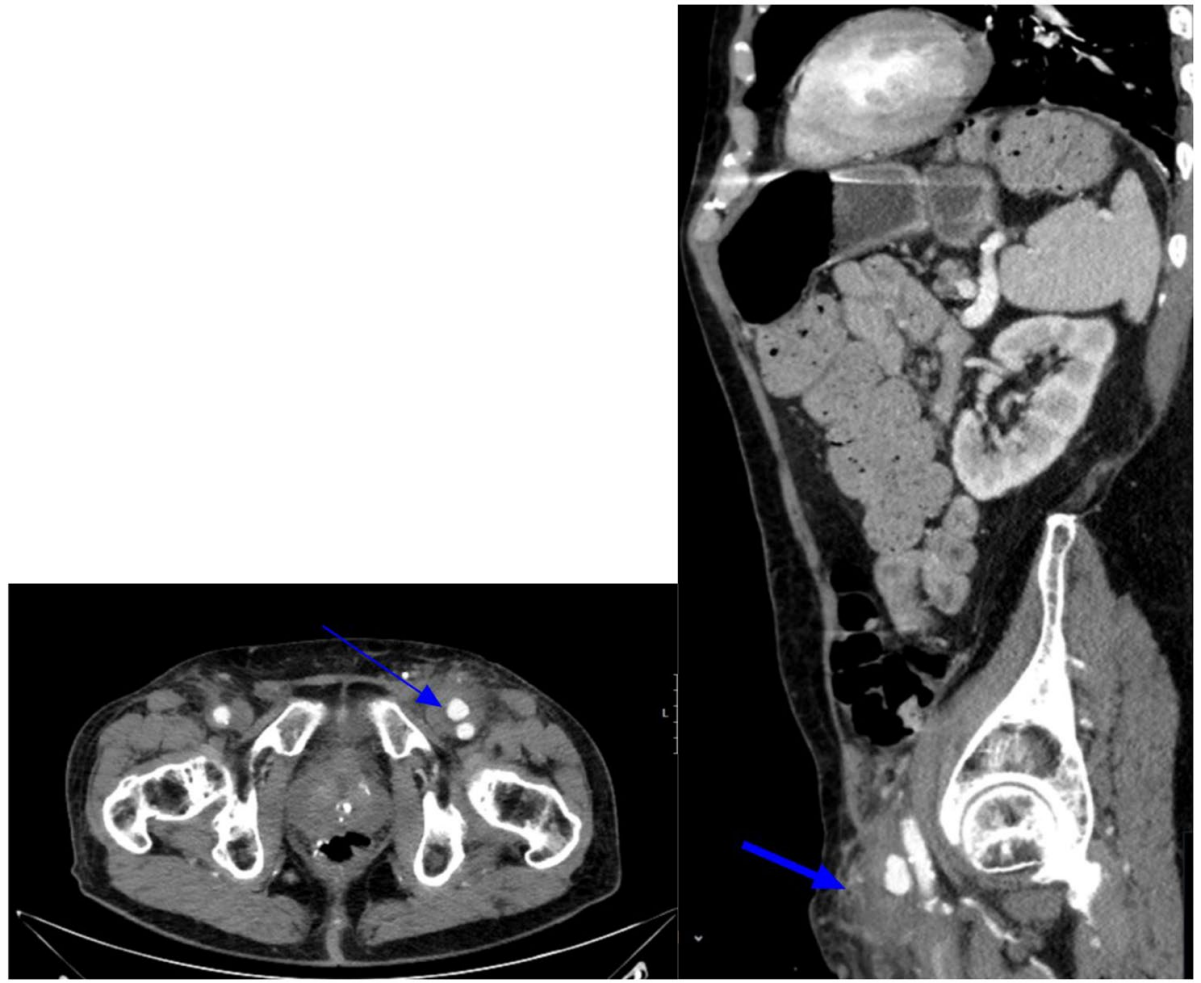
88 year old male with history of cerebral vascular accident presenting with nausea and vomiting. CT abdomen and pelvis in soft tissue window in Axial (**A**) and sagittal (**B**) reformats demonstrate a 1.4 cm hyperdensity (blue arrow) anteriorly at the left common femoral artery, most consistent with a pseudoaneurysm and heterogeneous density surrounding left common femoral vessels, suggestive of hematoma

When it came to abdominal imaging, these included 1 gastrointestinal bleed and 4 access site complications: 1 access site hematoma, 1 case of necrotizing fasciitis (Figs. 6), 1 pseudoaneurysm (Figs. 7) and 1 femoral artery thrombus. Most commonly, these patients reported some form of localized groin (swelling, infection, bleeding) or leg symptoms (leg pain, swelling). However, some presented with non-specific signs such as GI discomfort (abdominal pain, nausea, vomiting, diarrhea) or weakness. Of note, 1 case of pneumonia was also found on abdominal imaging.

The most common imaging finding from cardiac imaging (ECHO) was heart failure. There were 8 total cases in this population. The most common patient presentation was shortness of breath, which was found in 5 of 8 patients. Other presenting symptoms included hypotension, fatigue, abnormal ICD firing, chest pain, and hyponatremia.

In imaging of the extremities such as duplex ultrasounds (*n* = 40) and extremity MRI (*n* = 1), common imaging findings include 1 deep vein thrombosis and 4 access site complications: 3 cases of access site hematoma and one access site thrombus. These patients commonly presented with localized leg swelling or pain.

Ultimately, while echocardiograms and duplex extremity vasculature ultrasounds were the most abundant types of studies ordered with 47 and 40 studies respectively, CTA chest and echocardiograms were the 2 study modalities that most commonly resulted in acute findings of significance with 12 and 8 findings respectively (Table 5).

**Table 5:** Imaging findings in commonly imaged body locations*

CHEST IMAGING (*n* = 55)	
Pneumonia	7
Pleural effusion	4
Pulmonary embolism	1
Pulmonary hemorrhage	1
Pulmonary edema	1
Aortic aneurysm	1
Hematoma around ascending aorta	1
No acute findings	40
% of imaging with acute findings	27%
HEAD/NECK IMAGING (*n* = 54)	
Stroke	4
Brain hematoma	1
C-spine narrowing	1
No acute findings	48
% of imaging with acute findings	11%
ABDOMEN IMAGING (*n* = 49)	
Access site complication	4
GI bleed	2
Diverticulitis	1
Abdominal wall hematoma	1
Cystitis	1
Pneumonia	1
Pleural effusion	1
No acute findings	39
% of imaging with acute findings	20%
CARDIAC IMAGING (*n* = 47)	
Heart failure	8
No acute findings	39
% of imaging with acute findings	17%
EXTREMITY IMAGING (*n* = 41)	
Access site complication	4
Deep vein thrombosis	1
Decreased foot perfusion	1
No acute findings	35
% of imaging with acute findings	15%
MISCELLANEOUS IMAGING (*n* = 4)	
No acute findings	4

*Some imaging studies resulted in multiple acute findings

## Discussion

This study aims to report on trends in multi-modality emergency department cross-sectional imaging in the post-TAVR patient population within 30 days of their TAVR procedure. Among the 146 patients included in this study, there were 168 ED visits where 22 patients had 2 visits within this time span. While the most prevalent presenting symptoms were respiratory symptoms, mentioned in 45 of the 168 total visits (27%), and neurological symptoms, reported in 38 of the 168 total visits (23%), the most common acute findings of this patient population were heart failure (5.5%), pneumonia (5.5%), access site complications (5.5%), cerebrovascular events (3.4%) and pleural effusions (3.4%). Furthermore, while respiratory and neurological symptoms prevailed as the most common categories of presenting symptoms, neurologic and vascular indications prevailed as the most common categories for imaging indication. Thus, common presenting symptoms do not necessarily translate to common indications for imaging in this patient population.

This study also found that CTs and ultrasounds were the 2 most commonly utilized cross-sectional imaging modalities in the post-TAVR patient population. While echocardiograms and duplex extremity vasculature ultrasounds were the most abundant types of studies ordered with 47 and 40 studies respectively, CTA chest and echocardiograms were the 2 study modalities that most commonly resulted in acute findings of significance with 12 and 8 findings respectively. Echocardiograms consistently pinpointed heart failure symptoms and complications. Chest CT angiograms, from 26 scans, yielded significant findings in 12 patients, 10 of whom had pulmonary findings including but not limited to pneumonia and pleural effusions, and 2 of whom had aortic findings such as an aortic aneurysm or a hematoma surrounding the aorta. This reinforces the vital role of these imaging techniques in identifying key post-operative complications of patients who have undergone TAVR.

Similar to existing literature, this study found that readmissions within 30 days of the index admission were more likely due to non-cardiac related causes rather than cardiac-related causes^7,8^. Kolte et al. analyzed data from the 2013 US NRD and showed an all-cause readmission rate of 17.9%, with only 38.2% of readmissions due to cardiovascular causes. The authors noted that there was a median cost of $8,302 per patient for post-TAVR 30-day readmissions. In their study, heart failure was also the most common cause of all‐cause readmission in 22.5% of the cases.^7^ Comparably, Zahid et al.^9^. found 3.6% of 30‐day readmissions attributed to heart failure. Other studies also found that congestive heart failure and arrhythmias were the most common cardiac causes of 30-day readmission.^10^

Among non-cardiac causes, respiratory, infectious, bleeding and vascular conditions have been identified as the most common causes of 30-day readmission. This is comparable to our results where pneumonia (5.5%) and access site complications (5.5%) were the top non-cardiac findings within this patient population. In a study by Arora et al.^11^, they showed that the cost of an average 30-day TAVR readmission was $10,068, with no significant differences in 2012-17while the cost burden of readmissions for every TAVR performed was $1,883 in 2017, which had significantly declined from $4,061 in 2012.

Examining TAVR readmission trends as a whole, it seems that despite all-cause readmissions decreasing significantly over the years (2015–2018), 30‐day HF (heart failure) readmissions after TAVR remained steady. Moreover, HF readmissions were associated with increased mortality and resource use compared with non-HF readmission^9^. Given each HF readmission within 30 days is associated with an average increased cost of $13,000 more than for each non-HF readmission, focus on early recognition and complication prevention of HF in this post-operative patient population continues to be crucial^9^. Because HF readmission mortality has remained relatively unchanged over the years^9^, a low threshold for advising further testing and imaging for HF will likely benefit many patients. Methods such as suggesting echocardiograms and correlation with clinical data when signs of HF are apparent on cross-sectional imaging may be helpful. We also advocate for further research in developing post discharge interventions for this cohort to help mitigate the risk of readmissions and consequently reduce overall costs.

While not the most common acute imaging finding in in this study, post procedural strokes within the post procedural 30-day period are also prevalent at 2.7% of acute imaging findings. In the PARTNER trial, which included the continued access registry with 2621 patients, within 30 days of TAVR, 87 (3.3%) patients experienced a stroke, 85% of which were within 1 week of the procedure^12^. The most common presenting symptom for stroke in our study population was altered mental status, which resulted in a diagnosis of stroke in 4 of 6 patients that presented with this symptom. Given these statistics, it is important for radiologists to recognize this trend and be especially vigilant about signs of stroke in the immediate post-procedural timeframe.

In the post-op setting of TAVR procedures, the potential diagnoses of stroke and heart failure (HF) – both associated with diminished one-year survival rates^11^ – necessitate increased diagnostic vigilance when patients present to the ED. Among the scans conducted in our cohort, non-contrast CT or MRI brain was instrumental in diagnosing cerebrovascular events. Of the 39 head CTs, 4 revealed critical findings such as strokes and a brain hematoma while 1 of 4 head MRIs revealed a stroke. Echocardiography stood out as the definitive modality for diagnosing heart failure, with 8 out of 47 echocardiograms revealing acute results. For clinicians, recognizing the urgency of timely imaging and diagnosis is vital, and the choice of imaging technique should be informed by the most likely clinical in conjunction the ACR Appropriateness Criteria guidelines.

There are a few limitations in our study. First, the study is retrospective in nature. Secondly, the study comprises patients from a single institution which limits the sample size of the study, which may not be representative of broader TAVR populations, particularly those in other healthcare settings or geographical locations. Thirdly, the accuracy of our findings is contingent upon the accuracy of diagnostic coding and imaging reports, which can be variable across institutions. Additionally, while our study underscores the prevalence of certain imaging modalities, it does not necessarily elucidate the underlying reasons for the choice of one modality over another. It is also worth noting that, while we compared our findings with prior research, differences in patient selection criteria, study designs, and timeframes might account for some discrepancies in results.

## Conclusion

In conclusion, this research underscores the importance of early and precise imaging in diagnosing and managing post-TAVR patients within 30 days of their index admission. Future research is warranted to develop post-discharge interventions that can potentially reduce readmission rates and the associated healthcare costs. Given the results of our study, it is apparent that emergency radiologists play a crucial role in the diagnostic evaluation of acute presentations in this patient population. As the utilization of transcatheter approaches to aortic valve replacement increases with the aging population and emergency visits post-TAVR remains high, emergency radiologists need to be aware of the common imaging presentations in this post-procedural population as well as recommend appropriate followup imaging. Ultimately, proper imaging guidance can ensure that post-TAVR patients receive timely and effective care, thereby improving their overall outcomes.
